# Protective Effects Assessment of Combined Extracts from *Periplaneta americana* Residues and *Cybister chinensis* Motschulsky on Feline Renal Cells: In Vitro Evidence Related to Inflammation, Oxidative Stress, and Fibrosis

**DOI:** 10.3390/vetsci13040317

**Published:** 2026-03-26

**Authors:** Yue Sun, Canhui Hong, Yang Li, Zhengze Zhang, Futing Tan, Zhihuan Li, Wangling Qian, Yihao Che, Zhibin Yang

**Affiliations:** 1 Yunnan Provincial Key Laboratory of Entomological Biopharmaceutical R&D, College of Pharmacy, Dali University, 22 Wanhua Road, Xiaguan Town, Dali 671000, China; 15887944980@163.com (Y.S.); hongch09@163.com (C.H.); 18225840998@163.com (Y.L.); 18287721627@163.com (Z.Z.); 17869025994@163.com (F.T.); 17787501426@163.com (Z.L.); 13408793915@163.com (W.Q.); 2 National-Local Joint Engineering Research Center of Entomoceutics, Dali 671000, China

**Keywords:** feline chronic kidney disease, *Periplaneta americana* residue extract, *Cybister chinensis* extract, inflammation, oxidative stress, fibrosis

## Abstract

With the rising prevalence of feline kidney diseases, effective preventive and therapeutic strategies are urgently needed. This study evaluated the effects of *Cybister chinensis* extracts (CCME) and *Periplaneta americana* residue extracts (PAE) on inflammation-associated, oxidative stress-related, and fibrosis-related responses in Crandell-Rees Feline Kidney (CRFK) cells. Using MTT assays, flow cytometry, and qPCR, we assessed cytoprotection in models of lipopolysaccharide (LPS)-, hydrogen peroxide (H_2_O_2_)-, and palmitic acid (PA)-induced injury. Preliminary HPLC fingerprint analysis of three batches of a combined extract from *Periplaneta americana* residues and *Cybister chinensis* Motschulsky (CPCE) revealed similar chromatographic profiles, indicating good batch-to-batch consistency. Within non-cytotoxic ranges, CPCE increased cell viability and reduced apoptosis in injured CRFK cells. Anti-inflammatory effects were evidenced by significant downregulation of TNF-α and IL-6 mRNA. Potential antioxidant-related effects were suggested by decreased expression of oxidative stress–responsive genes SOD1, CAT, and GSTP1. In the PA model, anti-fibrotic potential was supported by reduced TGFB1 expression, accompanied by improvements in inflammatory and oxidative stress markers, and by decreased levels of fibrosis-associated markers α-SMA, COL I, and HCB III. These findings suggest that CPCE exerts cytoprotective effects in vitro, potentially through modulation of inflammation, oxidative stress, and fibrosis.

## Simple Summary

Feline chronic kidney disease is common in middle-aged and senior cats, causing progressive kidney damage through inflammation, oxidative stress, and fibrosis, with limited effective treatments. This study aimed to explore whether a combined extract from *Periplaneta americana* residues and *Cybister chinensis* Motschulsky could protect cat kidney cells. Using cat kidney cells, we tested the extract against three types of damage linked to kidney disease. Results showed the extract boosted cell survival, reduced inflammation and cell death, eased oxidative stress, and slowed fibrosis-related changes. These findings suggest the extract could be developed into functional pet foods or veterinary products to help prevent or manage feline kidney disease, offering a new natural option for cat health care.

## 1. Introduction

As the population of pets continues to expand, there is a growing focus on the prevention and management of chronic diseases in aging companion animals, with particular emphasis on felines [1,2]. Concurrently, chronic kidney disease (CKD) has emerged as one of the most prevalent chronic conditions among middle-aged and senior felines, characterized by a progressive decline in renal structure and function [3]. CKD particularly kidney failure and urinary tract infections, are especially common in middle-aged and senior cats [4]. Inflammatory response, oxidative stress, and fibrosis are the primary mechanisms of renal injury, and they interact in a complex manner, ultimately leading to the destruction of renal structure and function [3,5,6]. Therefore, the ability to suppress inflammation, reduce oxidative damage, and slow down fibrosis during the progression of kidney diseases in cats is of significant clinical importance for renal protection and functional recovery. CRFK cells represent an immortalized feline kidney cell line frequently utilized in vitro models [7]. These models notably include pro-fibrotic responses associated with TGF-β1/Ang II and cellular damage related to oxidative stress [8,9].

Kidney diseases in cats often present with mild early symptoms, making them difficult to detect. Although many treatments are available to help alleviate the symptoms of kidney failure and slow the progression of the disease, current treatment options still have numerous drawbacks and limitations, including an inability to cure the condition, high treatment costs, potential side effects of medications, challenges in dietary therapy, and issues with the widespread adoption of emerging treatments [10,11]. How to effectively prevent and treat kidney diseases in cats has become a key issue that needs to be addressed in pet health management [12].

Inflammatory responses and apoptosis are key factors in CKD progression. LPS activates the TLR4/NF-κB pathway, resulting in the release of inflammatory cytokines such as *TNF-α* and *IL-6*, thereby intensifying renal inflammation [13], In parallel, LPS-induced upregulation of pro-apoptotic proteins such as *BAX* and *Bid* enhances apoptosis of renal tubular cells, accelerating renal damage. The interplay between inflammation and apoptosis establishes a positive feedback loop, further aggravating CKD progression [14]. Oxidative damage is a major contributor to CKD. Hydrogen peroxide, by generating excessive reactive oxygen species (ROS), disrupts the antioxidant defense system, activates inflammatory responses, impairs mitochondrial function, and induces apoptosis, thereby initiating oxidative stress in CRFK cells [13,15]. In addition, PA, a common free fatty acid (FFA) and dietary saturated fat, contributes to the intracellular accumulation of lipids at high concentrations, leading to lipotoxicity. Such lipotoxicity has been shown to cause mitochondrial dysfunction, excessive production of ROS, and the activation of inflammatory and apoptotic pathways. PA induces apoptosis in podocytes, glomerular mesangial cells, and renal tubular epithelial cells through multiple mechanisms, including mitochondrial and endoplasmic reticulum (ER) stress. The combined effects of oxidative stress, ER stress, and apoptosis collectively promote tubulointerstitial fibrosis [16,17,18].

Preparations derived from *Periplaneta americana* are extensively employed in clinical practice, particularly in the form of bioactive extracts such as Kangfuxin. The industrial production of these preparations results in significant ethanol-extracted residues, which serve as a reliable and stable source of raw materials that can be effectively repurposed through energy-efficient recycling strategies [19,20,21,22,23]. Previous phytochemical studies have shown that *Periplaneta americana* extracts contain multiple reported bioactive constituents, including polyols, peptides, polysaccharides, steroids, terpenes, alkaloids, flavonoids, and isocoumarins, which may collectively contribute to their pharmacological activities [24,25,26,27]. Recent studies have highlighted that ethanol-extracted residues of *Periplaneta americana* retain significant quantities of proteins, polypeptides, and other bioactive compounds. Furthermore, the incorporation of these residues into animal diets has been demonstrated to enhance immune function and antioxidant capacity without negatively impacting growth performance, suggesting the preservation of substantial biological activity. Consequently, PAE may hold value not only as a refined extract but also as a source of insect-derived functional ingredients with potential anti-inflammatory, antioxidant, and tissue-repairing properties. This warrants further pharmacological investigation and exploration of high-value applications [28,29,30].

As an aquatic insect, *Cybister chinensis* Motschulsky has garnered interest due to its potential bioactive compounds and ecological significance [31,32]. Research on *Cybister chinensis Motschulsky*’s bioactive components is limited and evolving [33]. Compared with Periplaneta americana, the chemical basis of *Cybister chinensis* Motschulsky is less well characterized. According to the currently available literature, its extract may contain fatty acids and other small bioactive molecules with antioxidant and anti-inflammatory potential, although the evidence remains limited and requires further validation [34,35,36,37]. However, these findings are indirect and require validation for *Cybister chinensis* Motschulsky. While some bioactivities of *Cybister chinensis* Motschulsky extracts have been observed [33], the underlying mechanisms are unclear, and conclusions are preliminary. The extracts from *Cybister chinensis* Motschulsky and *Periplaneta americana* are rich in various bioactive components, showing significant potential for anti-inflammatory, antioxidant, and anti-fibrotic effects [33,38]. Although these extracts have shown promising application prospects in the treatment of various diseases, their research in feline renal diseases remains limited [33,38,39,40,41,42]. Existing studies provide preliminary evidence for their potential in veterinary medicine, particularly in the potential application value of functional foods and pharmaceuticals for cats, which warrants further in-depth exploration.

**Figure 1:**
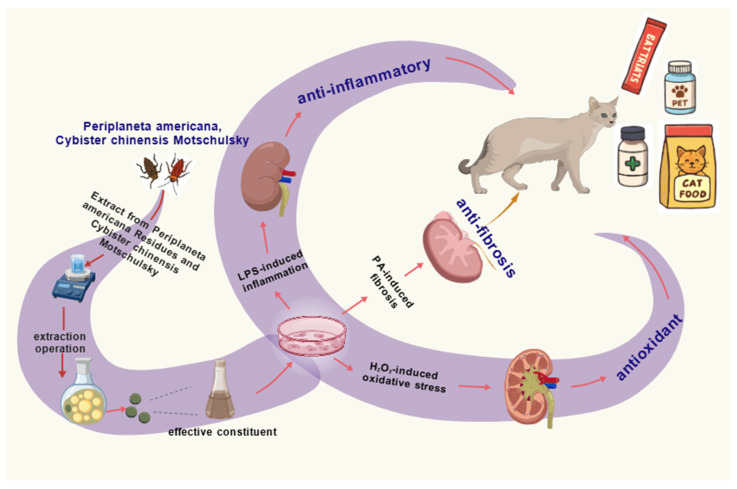
Overview of the in vitro experimental design for evaluating the effects of CPCE on inflammation-, oxidative stress-, and fibrosis-related responses in CRFK cells, and its potential applications.

In this study, we combined PAE and CCME to develop a natural composite extract for the protection of feline renal cells. The rationale for this combination was based on the potentially complementary bioactivities of the two extracts, with anti-inflammatory and tissue repair–related constituents in PAE potentially complementing the antioxidant peptides, high-quality proteins, and minerals in CCME. However, direct evidence for synergy or superiority over single-extract treatment was not established in the present study. To validate the hypothesis, CRFK cells were employed to characterize the cytoprotective effects of the combined extracts by profiling endpoints related to inflammatory signaling, oxidative stress, and fibrosis-associated processes. MTT assays, flow cytometry, and RT-qPCR were utilized to assess and compare the protective effects against injuries induced by LPS, H_2_O_2_, and PA challenges. Therefore, this study aimed to investigate the protective effects of combined extracts from *Periplaneta americana* residues and *Cybister chinensis* on feline renal cells (CRFK), with a particular focus on anti-inflammatory, oxidative stress–related, and fibrosis-associated mechanisms in vitro. Overall, this study provides experimental evidence supporting the development of functional cat foods and pharmaceuticals for preventing and treating feline kidney diseases, and offers a rationale for incorporating insect-derived extracts as functional ingredients in pet nutrition and therapeutics (Figure 1).

## 2. Materials and Methods

### 2.1. Materials and Chemicals

The *Periplaneta americana* residue was obtained from Good Doctor Pharmaceutical Group Co., Ltd., Chengdu, China, and the *Cybister chinensis* Motschulsky was purchased from Huatang Biotechnology Co., Ltd. in Bozhou City, Bozhou, China.

The CRFK cells line was purchased from Wuhan Procell Life Science Technology Co., Ltd., Wuhan, China. LPS was purchased from Sigma-Aldrich, St. Louis, MO, USA. PA was purchased from Macklin, Shanghai, China. Fatty-acid-free bovine serum albumin (BSA) was purchased from Biosharp, Hefei, China. 3% H_2_O_2_ solution was obtained from Sigma-Aldrich, St. Louis, MO, USA. The SteadyPure RNA Extraction Kit, Evo-M-MLV Reverse Transcription Kit, and SYBR Green Pro-Taq HS q-PCR Kit were sourced from Hunan Ai Rui Biotechnology Co., Ltd., Changsha, China. The Annexin-V/FITC Cell Apoptosis Detection Kit was obtained from Proteintech, Wuhan, China. The Cat α-SMA ELISA Kit, Cat COL I ELISA Kit, and Cat HCBIII ELISA Kit were purchased from Jiangsu Jingmei Biotechnology Co., Ltd., Jiangsu, China. 5 mL Flow Tubes were purchased from Becton, Dickinson and Company, Franklin Lakes, NJ, USA. Flow cytometric analysis was performed using a FACSCanto II flow cytometer (Becton Dickinson, Franklin Lakes, NJ, USA).

### 2.2. Periplaneta americana Residue and Cybister chinensis Motschulsky Ethanol Extracts

#### 2.2.1. Preparation of *Periplaneta americana* Residue Extracts

A total of 12 g of *Periplaneta americana* residue powder was placed in a round-bottom flask. 75% ethanol solution was added at a sample-to-solvent ratio of 1:20 (g/mL). The mixture was heated in a water bath at 90 °C, and solid–liquid separation was performed every 2 h. This process was repeated until the supernatant became nearly colorless. The combined filtrates were concentrated by rotary evaporation and then freeze-dried for 48 h.

Based on the analysis, the extraction yield of *Periplaneta americana* residue was found to be 7.68%. Finally, the extract was stored at −80 °C for future use.

#### 2.2.2. Preparation of *Cybister chinensis* Motschulsky Extracts

Dried *Cybister chinensis* Motschulsky (5 kg) was ground in a pulverizer until the material was fully powdered. The powdered material was then collected and divided into two portions of 2.5 kg each, placed into 50 L containers. A 75% ethanol solution was added at a sample-to-solvent ratio of 1:10 (g/mL), and the mixture was soaked for one week, with stirring performed three times daily. After one week of soaking, the mixture was filtered, and the residue was subjected to two additional extractions following the same procedure. The filtrates from all three extractions were combined and concentrated by rotary evaporation and freeze-dried [33].

Based on the analysis, the extraction yield of *Cybister chinensis* Motschulsky was found to be 24.95%. The extract was stored at −80 °C for future use.

#### 2.2.3. HPLC Fingerprint Analysis of CPCE for Batch Consistency Evaluation

##### Preparation of Sample Solutions for HPLC Fingerprint Analysis

Three batches of CPCE were used for HPLC fingerprint analysis. For each batch, 20 mg of the sample was accurately weighed and dissolved in 1 mL of 90% methanol aqueous solution. The mixture was placed into an ultrasonic bath for 20 min. After ultrasonic extraction, the solution was centrifuged, and the supernatant was collected. The supernatant was filtered through a 0.22 μm membrane filter and transferred into an HPLC vial for analysis.

##### HPLC Fingerprint Analysis

Chromatographic analysis was performed using an HPLC system. The analysis was carried out at 25 °C on a ZORBAX SB-C18 column (4.6 × 250 mm, 5 μm). The mobile phase consisted of A (water with 0.1% TFA) and B (methanol with 0.1% TFA). For the preparation of the mobile phases, 1.0 mL trifluoroacetic acid (TFA) was added to 1 L of ultrapure water and 1 L of chromatographic-grade methanol, respectively. Both mobile phases were filtered through a 0.22 μm membrane filter and sonicated for 20 min before use.

The gradient program was as follows: 0–60 min, 95–5% A (5–95% B); 60–70 min, 5% A (95% B). The UV absorbance was monitored at 280 nm, and the solvent flow rate was kept at 1.0 mL/min. The sample concentration was 1 mg/mL, and the injection volume was 10 μL.

#### 2.2.4. Preparation of Extract Stock Solutions and Working Concentrations

For each extract, 30 mg of the PAE and 30 mg of the CCME were fully dissolved in phosphate-buffered saline (PBS) to prepare stock solutions at a concentration of 5 mg/mL. The stock solutions were aliquoted (100 μL per tube) and stored at −20 °C until use. In the cell-based experiments, the stock solutions were diluted using a complete culture medium, and the two extracts were administered in combination, referred to as CPCE, which represents the combination of PAE and CCME. In all combination treatments, PAE and CCME were introduced at equal concentrations, resulting in final concentrations of 0.625, 1.25, 2.5, and 5 μg/mL for each extract (i.e., 0.625–5 μg/mL PAE + 0.625–5 μg/mL CCME in the respective groups). In all cell-based experiments involving CPCE, PBS-matched vehicle controls were included. Specifically, the control and model groups received volumes of PBS equivalent to the total stock-solution volume added to the corresponding CPCE-treated wells, so that any potential effect of the PBS vehicle was controlled for in parallel.

### 2.3. Anti-Inflammatory Activity of CPCE on LPS-Induced CRFK Cells Damage

**Figure 2:**
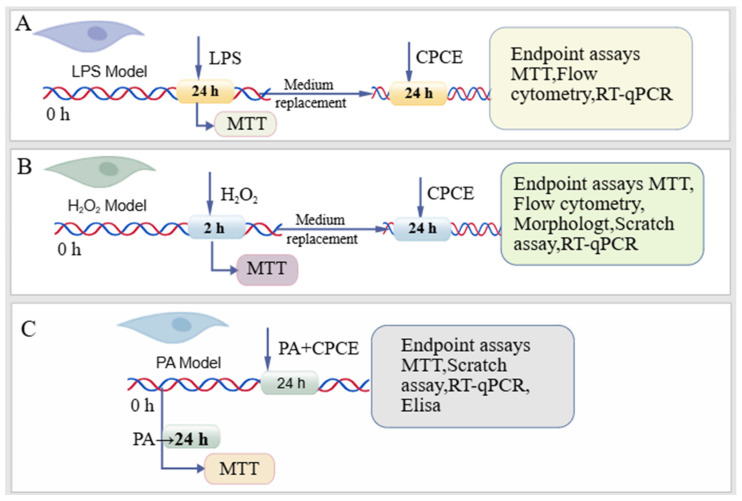
Schematic summary of the experimental designs described in Section 2.3, Section 2.4 and Section 2.5 for the LPS-, H_2_O_2_-, and PA-induced injury models in CRFK cells. (**A**) CRFK cells were treated with LPS for 24 h, then treated with CPCE for 24 h, followed by MTT assay, Flow cytometry, and RT-qPCR. (**B**) CRFK cells were treated with H_2_O_2_ for 2 h, then treated with CPCE for 24 h, followed by MTT assay, morphological observation, Flow cytometry, scratch assay, and RT-qPCR. (**C**) CRFK cells were treated with PA alone for 24 h for MTT assay, or treated with PA and CPCE for 24 h, followed by MTT assay, scratch assay, RT-qPCR, and ELISA.

The overall experimental timelines for the LPS-induced injury models are summarized in Figure 2.

#### 2.3.1. Effect of LPS and CPCE on CRFK Cells Viability

CRFK cells were digested using trypsin containing 0.25% EDTA, collected, and resuspended to a concentration of 1 × 10^5^ cells/mL. The cells were seeded into a 96-well plate, and after adherence, the culture medium was discarded. The cells were washed with PBS and treated with varying concentrations of CPCE (20, 40, 80, 160, 320, 640, and 1280 μg/mL) and LPS (60, 80, 100, 120, 140, 160, and 180 μg/mL). A blank control group was set up (0 μg/mL PAE and CCME and 0 μg/mL LPS). The plates were incubated at 37 °C with 5% CO_2_ for 24 h. Subsequently, 20 μL of MTT solution was added to each well, and the plates were incubated for an additional 4 h. The supernatant was then discarded, and 150 μL of DMSO was added to each well. The wells were shaken at 80 rpm for 5 min, and absorbance at 490 nm was measured using a microplate reader (Varioskan LUX, Thermo Scientific, Waltham, MA, USA) to assess cell viability.

#### 2.3.2. Effect of CPCE on LPS-Induced CRFK Cells Proliferation

CRFK cells were harvested and subsequently seeded into a 96-well plate at a density of 1 × 10^5^ cells/mL. Following cell adherence, the culture medium was removed. The cells were then categorized into three experimental groups: a control group, an LPS group (treated with 160 μg/mL lipopolysaccharide), and CPCE treatment groups, in which cells were first exposed to 160 μg/mL LPS for 24 h and then treated with CPCE at 1.25, 2.5, and 5 μg/mL for an additional 24 h after medium replacement. The cells were incubated for an additional 24 h, after which cell proliferation was evaluated using the MTT assay.

#### 2.3.3. Detection of Cell Apoptosis by Flow Cytometry

CRFK cells were cultured in 6-well plates and subjected to LPS-induced injury followed by CPCE treatment to assess apoptosis. Cells were detached using trypsin without EDTA, and the enzyme was promptly aspirated to prevent excessive trypsinization. The resulting cell suspension, along with the supernatant, was collected and centrifuged at 1000 rpm for 3 min. The cell pellets were then resuspended in 100 μL of binding buffer and incubated with 5 μL of Annexin V-FITC in the dark at room temperature for 5 min, followed by staining with 5 μL of propidium iodide (PI). Subsequently, the cells were resuspended in 400 μL of PBS and analyzed using a flow cytometer.

For each sample, a minimum of 10,000 events were collected, and three independent biological replicates (*n* = 3) were subjected to analysis. Compensation was established using unstained, Annexin V-FITC single-stained, and PI single-stained controls. Initial gating was performed using forward scatter (FSC) and side scatter (SSC) to exclude debris and non-cellular events. Apoptotic cell populations were then quantified by quadrant analysis based on Annexin V-FITC and PI staining. Annexin V^+^/PI^−^ cells were defined as early apoptotic cells, Annexin V^+^/PI^+^ cells as late apoptotic cells, Annexin V^−^/PI^−^ cells as viable cells, and Annexin V^−^/PI^+^ cells as necrotic cells. The total apoptosis rate was calculated as the sum of early and late apoptotic cells. Flow-cytometry data were analyzed by an investigator blinded to group allocation. The compensation matrix and gating strategy for the LPS-induced apoptosis assay are provided in Supplementary Table S1 and Supplementary Figure S1, respectively.

#### 2.3.4. Real-Time Quantitative PCR to Detect mRNA Expression Levels of Inflammatory and Apoptosis-Related Genes in Cells

CRFK cells were cultured in 6-well plates and subjected to treatment with LPS in addition to CPCE. Subsequently, mRNA expression levels were quantified utilizing the Axygen kit, (Hunan Aikerui Bioengineering Co., Ltd., Changsha, China). RNA concentration and purity were determined by measuring UV absorbance with a NanoDrop ND-2000 spectrophotometer (Life Real, Hangzhou, China).

Reverse transcription was conducted utilizing the Evo M-MLV Reverse Transcription Premix Kit in accordance with the manufacturer’s protocol for quantitative PCR (qPCR).

This procedure utilizes the AccuRea SYBR Green Pro Taq HS Premix qPCR Kit (Hunan Aikerui Bioengineering Co., Ltd., Changsha, China). Primers were synthesized according to the sequences in Table 1, and qPCR reaction system and procedures were prepared according to the strict instructions in the manual (Bio-Rad, Hercules, CA, USA), PCR reactions were performed using the cDNA obtained from the previous reverse transcription step (APExBio, Houston, TX, USA), and the corresponding primer information is provided in Table 1.

After the completion of the RT-PCR reaction, data were collected and analyzed. GAPDH was used as the internal reference gene, and the results were calculated using the 2^−△△CT^ method. Each reaction was performed in triplicate for biological repeats.

### 2.4. Effects of CPCE on H_2_O_2_-Induced CRFK Cell Injury and Oxidative Stress-Related Responses

The overall experimental timelines for the H_2_O_2_-induced injury models are summarized in Figure 2.

#### 2.4.1. Effect of H_2_O_2_ on CRFK Cells Viability

CRFK cells were seeded into 96-well plates at 1 × 10^5^ cells/mL. After attachment, the medium was removed and cells were rinsed with PBS. Cells were then treated with H_2_O_2_ at 0, 0.25, 0.5, 1.0, 2.0, 4.0, 8.0, 16.0, 32.0, or 64.0 mM; a blank control (0 mM H_2_O_2_) was included. Cultures were maintained at 37 °C in 5% CO_2_ for 2 h, after which cell viability/proliferation was evaluated by the MTT assay.

#### 2.4.2. Effect of CPCE on Proliferation of H_2_O_2_-Induced CRFK Cells

CRFK cells were harvested and placed into a 96-well plate at a concentration of 1 × 10^5^ cells/mL. Once they adhered, the culture medium was removed. The cells were divided into three groups: the control group, the H_2_O_2_ group (1.0 mM H_2_O_2_), and the group treated with varying concentrations of CPCE (pre-treated with 1.0 mM H_2_O_2_ for 2 h, followed by the addition of 0.625, 1.25 and 2.5 μg/mL of CPCE). The MTT assay was employed to assess cell proliferation following a 24 h culture period.

#### 2.4.3. Effect of CPCE on the Morphology of H_2_O_2_-Induced CRFK Cells

The CRFK cells were cultivated in 12-well plates before being treated with H_2_O_2_ and CPCE. The cellular morphology was then examined using an inverted microscope (Motic China Group Co., Ltd., Xiamen, China).

#### 2.4.4. Detection of Cell Apoptosis by Flow Cytometry

Following H_2_O_2_ exposure and CPCE treatment, both floating and adherent CRFK cells were collected, stained with Annexin V-FITC/PI, and analyzed by flow cytometry to determine the apoptosis rate. The compensation matrix and gating strategy for the H_2_O_2_-induced apoptosis assay are provided in Supplementary Table S2 and Supplementary Figure S2, respectively.

#### 2.4.5. Scratch Assay to Assess Wound Closure in H_2_O_2_-Induced CRFK Cells

CRFK cells were seeded into 12-well plates at a density of 3 × 10^5^ cells/mL. Upon reaching approximately 80% confluence, a linear wound was created using a sterile 200 μL pipette tip. The culture medium was subsequently removed, and the wells were gently rinsed with PBS. The cells were then exposed to H_2_O_2_, either in the presence or absence of extracts derived from CPCE. The scratch assay was performed in serum-free medium, and cell proliferation was not experimentally suppressed. Cell migration was monitored for comparative analysis. Images were captured at 0 and 26 h under an inverted microscope (×10), and the same field was recorded for each well at both time points. Wound closure was quantified using ImageJ-win 64 (version 1.49; National Institutes of Health, Bethesda, MD, USA) based on wound area and calculated as: wound closure (%) = [(A 0 h − A 26 h)/A 0 h] × 100%, Three replicate wells were analyzed per group (*n* = 3).

#### 2.4.6. Real-Time Quantitative PCR to Assess mRNA Expression Levels of Antioxidant-Related Genes in Cells

CRFK cells were placed in 6-well plates and treated with H_2_O_2_ and extracts from PAE and CCME. Following this, mRNA expression levels were assessed using the Axygen kit, and the corresponding primer information is provided in Table 2.

### 2.5. Effects of CPCE on Fibrosis-Related Responses in PA-Induced CRFK Cells

The overall experimental timelines for the PA-induced injury models are summarized in Figure 2.

#### 2.5.1. Preparation of PA–BSA Stock Solution

To prepare the PA–fatty-acid–free BSA complex for subsequent cell treatment, a PA solution was initially prepared by dissolving 37.5 mg of PA in 10 mL of ddH_2_O within a 70 °C water bath, accompanied by gentle agitation, resulting in a 13.5 mM PA solution. Concurrently, a 30% fatty-acid–free BSA solution was prepared by dissolving 3 g of fatty-acid–free BSA in 10 mL of ddH_2_O in a 55 °C water bath. The PA and BSA solutions were then combined in a 1:1 volume ratio and incubated in a 55 °C water bath until complete dissolution was achieved. The resultant mixture was subsequently filtered through a 0.45 μm membrane filter to yield a 6.75 mM PA stock solution, which was stored at 4 °C until further use.

#### 2.5.2. Effect of PA on CRFK Cells Viability

CRFK cells were detached using 0.25% EDTA–trypsin, collected, and adjusted to a concentration of 1 × 10^5^ cells/mL prior to seeding into 96-well plates. Following cell attachment, the medium was aspirated, and the cells were rinsed with PBS. Subsequently, the cells were exposed to varying concentrations of PA (0, 100, 200, 300, 400, or 500 μM) and incubated at 37 °C in an atmosphere containing 5% CO_2_ for a duration of 24 h. Cell proliferation was then assessed utilizing the MTT assay.

#### 2.5.3. Effect of CPCE on the Proliferative Activity of CRFK Cells Under PA-Induced Damage

After collection, CRFK cells were seeded into 96-well plates at a density of 1 × 10^5^ cells/mL. The culture supernatant was discarded after the cells adhered. The cells were then treated according to the following groups: the control group, the model group (100 μM PA), and groups with different concentrations of the extracts PAE and CCME (1.25, 2.5, and 5 μg/mL) along with PA at the same concentration as the model group. Cell proliferation activity was assessed using the MTT assay after 24 h of co-culturing.

#### 2.5.4. Scratch Assay to Assess Wound Closure in PA-Induced CRFK Cells

CRFK cells were cultured in 12-well plates, and a scratch wound was created prior to the administration of PA, as well as CPCE. The scratch assay was performed in serum-free medium, and no proliferation-suppressing treatment was applied. Images were captured at 0 and 24 h under an inverted microscope (×10), and the same field was recorded for each well at both time points. Wound closure was quantified using ImageJ-win 64 based on wound area and calculated as: wound closure (%) = [(A 0 h − A 24 h)/A 0 h] × 100%, Three replicate wells were analyzed per group (*n* = 3).

#### 2.5.5. Real-Time Quantitative PCR to Assess mRNA Expression of Fibrosis-Related Gene TGFB1

CRFK cells were grown in 6-well plates and exposed to PA in combination with CPCE. After treatment, mRNA expression levels were measured using the Axygen kit, and the corresponding primer information is provided in Table 1, Table 2 and Table 3.

#### 2.5.6. Detection of α-SMA, COL I and HCB III Levels in PA-Induced CRFK Cells

CRFK cells were cultured in 6-well plates and treated with PA in combination with CPCE. After 24 h of treatment, the culture supernatants were collected and centrifuged to remove cell debris. The levels of α-SMA, collagen type I (COL I), and (HCBIII) were determined using commercial ELISA kits (Jiangsu Jingmei Biotechnology Co., Ltd., Yancheng City, China) according to the manufacturer’s instructions. The absorbance was measured using a microplate reader, and the concentrations were calculated based on the corresponding standard curves.

### 2.6. Statistical Analysis

All data are presented as mean ± SEM from at least three independent experiments. Normality of the data was evaluated using the D’Agostino and Pearson omnibus test. For comparisons among more than two groups, one-way analysis of variance (ANOVA) was conducted. When a significant overall difference was detected by ANOVA, Dunnett’s post hoc test was applied for multiple comparisons against the control or model group, as appropriate. A two-sided *p* value of <0.05 was considered statistically significant. Data analysis was performed using GraphPad Prism 9.3.1 (GraphPad Software, San Diego, CA, USA).

## 3. Results

### 3.1. HPLC Fingerprint Analysis of CPCE

**Figure 3:**
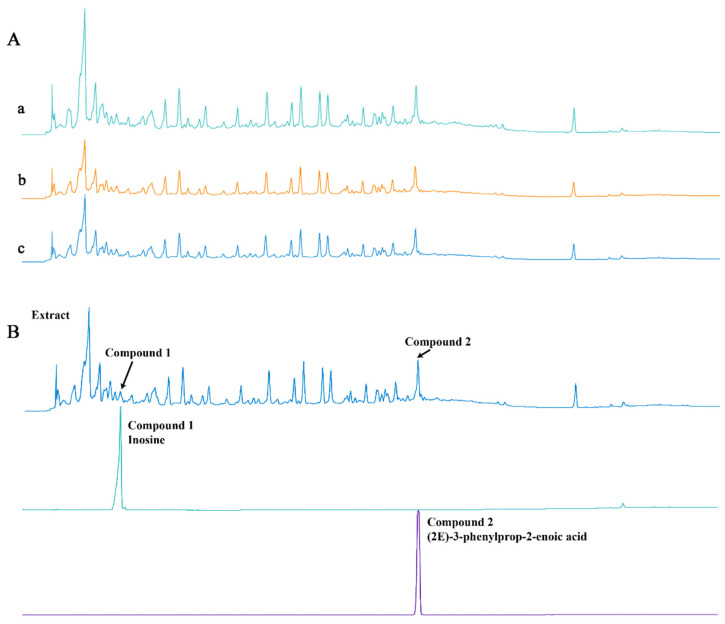
(**A**) HPLC chromatographic fingerprints of three independent batches of CPCE. a, Batch 1; b, Batch 2; c, Batch 3. (**B**) HPLC chromatographic fingerprints of extract, inosine and (2E)-3-phenylprop-2-enoic acid.

As shown in Figure 3A, the HPLC chromatographic fingerprints of the three independent batches of CPCE (Batch 1, a; Batch 2, b; Batch 3, c) were generally similar under identical chromatographic conditions. Most characteristic peaks appeared at comparable retention times across the three batches, and the overall chromatographic profiles showed similar peak distribution patterns. Although minor differences in relative peak intensity were observed, no obvious batch-dependent variation in the overall fingerprint profile was found. These results suggest acceptable batch-to-batch consistency of CPCE at the fingerprint level and provide preliminary compositional profiling information for the combined extract used in the present study. In addition, we identified two major compounds in the extract, namely inosine and (2E)-3-phenylprop-2-enoic acid, which were assigned based on comparative HPLC analysis with reference substances, as illustrated in Figure 3B.

### 3.2. Anti-Inflammatory Activity of CPCE on LPS-Induced CRFK Cells

#### 3.2.1. Effect of LPS on the Viability of CRFK Cells

**Figure 4:**
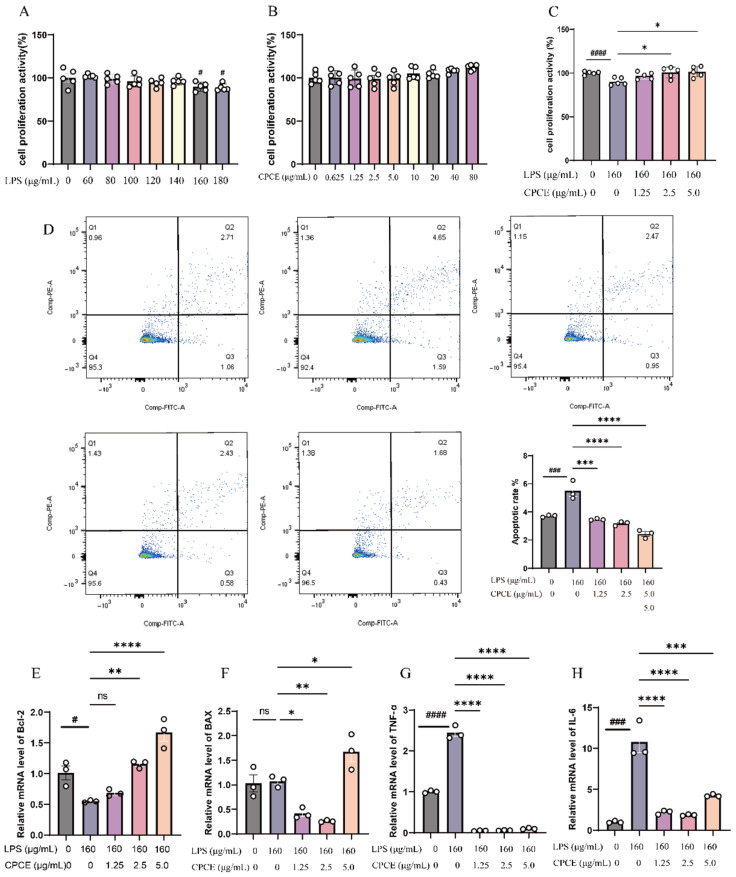
Anti-inflammatory activity of CPCE on LPS-induced CRFK cells. (**A**) Cell proliferation activity in the LPS-treated groups at various concentrations (*n* = 5). (**B**) Comparison of cell proliferation activity in CPCE at different concentrations (*n* = 5). (**C**) Effect of the Extracts of CCME and PAE on the survival rate of LPS-treated CRFK cells (*n* = 5). (**D**) Effect of CPCE on the apoptosis rate of LPS-induced CRFK cells (*n* = 3). (**E**–**H**) Effect of CPCE on the mRNA expression levels of apoptosis and inflammation-related genes *Bcl-2*, *Bax*, *TNF-α*, *IL-6* in LPS-induced CRFK cells (*n* = 3). ^#^ *p* < 0.05, ^###^ *p* < 0.001, ^####^ *p* < 0.0001 vs. control, * *p* < 0.05, ** *p* < 0.01, *** *p* < 0.001, **** *p* < 0.0001 vs. model.

Following 24 h of exposure to LPS in CRFK cells, the concentration of LPS significantly influenced cellular proliferative activity. In comparison to the negative control (0 μg/mL), cell proliferation was markedly diminished at concentrations of 160 and 180 μg/mL (*p* < 0.001). Consequently, a concentration of 160 μg/mL LPS was chosen as the working dose for the establishment of the LPS-induced inflammatory injury model accompanied by reduced cell viability in CRFK cells (see Figure 4A).

#### 3.2.2. Effect of CPCE on the Viability of CRFK Cells

As illustrated in Figure 4B, varying concentrations of the CPCE (10, 20, 40, 80, 160, 320, and 640 μg/mL) demonstrated a notable stimulatory effect on cell proliferation. However, at a concentration of 1280 μg/mL, CPCE significantly suppressed cell proliferation. Consequently, we opted to utilize CPCE at concentrations below 640 μg/mL for subsequent experiments.

#### 3.2.3. Effect of CPCE on LPS-Induced Proliferation of CRFK Cells

As illustrated in Figure 4C, the administration of 160 μg/mL of LPS markedly suppressed cell proliferation. In contrast, extracts from CPCE partially ameliorated this effect at specific concentrations. Notably, treatment with extract concentrations of 2.5 μg/mL and 5.0 μg/mL resulted in a statistically significant enhancement in cell viability relative to the model group (*p* < 0.01). These findings suggest that CPCE exerts a protective influence on the proliferative capacity of LPS-induced CRFK cells.

#### 3.2.4. Detection of Cell Apoptosis by Flow Cytometry

Figure 4D shows that the apoptosis rate in the LPS-treated group was significantly higher than in the control group (*p* < 0.001), indicating that the selected LPS concentration induced not only inflammatory activation but also cytotoxic/apoptotic responses. In contrast, treatment with CPCE at concentrations of 1.25, 2.5, and 5.0 μg/mL resulted in a significant reduction in the apoptosis rate of CRFK cells (*p* < 0.001), with the effect being dose-dependent. These findings suggest that specific concentrations of CPCE can effectively inhibit LPS-induced apoptosis in CRFK cells, with the highest concentration demonstrating the most pronounced effect.

#### 3.2.5. Effect of CPCE on the mRNA Expression Levels of Apoptosis and Inflammation-Related Genes in LPS-Induced CRFK Cells

As illustrated in Figure 4E–H, the LPS challenge in CRFK cells resulted in a significant downregulation of *Bcl-2* mRNA compared to the control group (*p* < 0.05), while Bax mRNA levels were upregulated. Concurrently, the expression of inflammatory markers *TNF-α* and *IL-6* was significantly elevated (*p* < 0.0001). Treatment with CPCE at varying concentrations led to a significant suppression of *TNF-α* and *IL-6* mRNA levels in comparison to the LPS group (*p* < 0.0001). Notably, treatment at concentrations of 2.5 and 5.0 μg/mL significantly increased *Bcl-2* mRNA levels (*p* < 0.01), with the exception of the 1.25 μg/mL group. Bax mRNA expression was significantly reduced in the 1.25 and 2.5 μg/mL groups (*p* < 0.05); however, this reduction was not evident at the 5.0 μg/mL concentration, where Bax expression surpassed that of the model group, suggesting a biphasic dose–response relationship.

### 3.3. Effects of CPCE on H_2_O_2_-Induced Injury and Oxidative Stress-Related Responses in CRFK Cells

#### 3.3.1. Effect of Different Concentrations of H_2_O_2_ on the Viability of CRFK Cells

**Figure 5:**
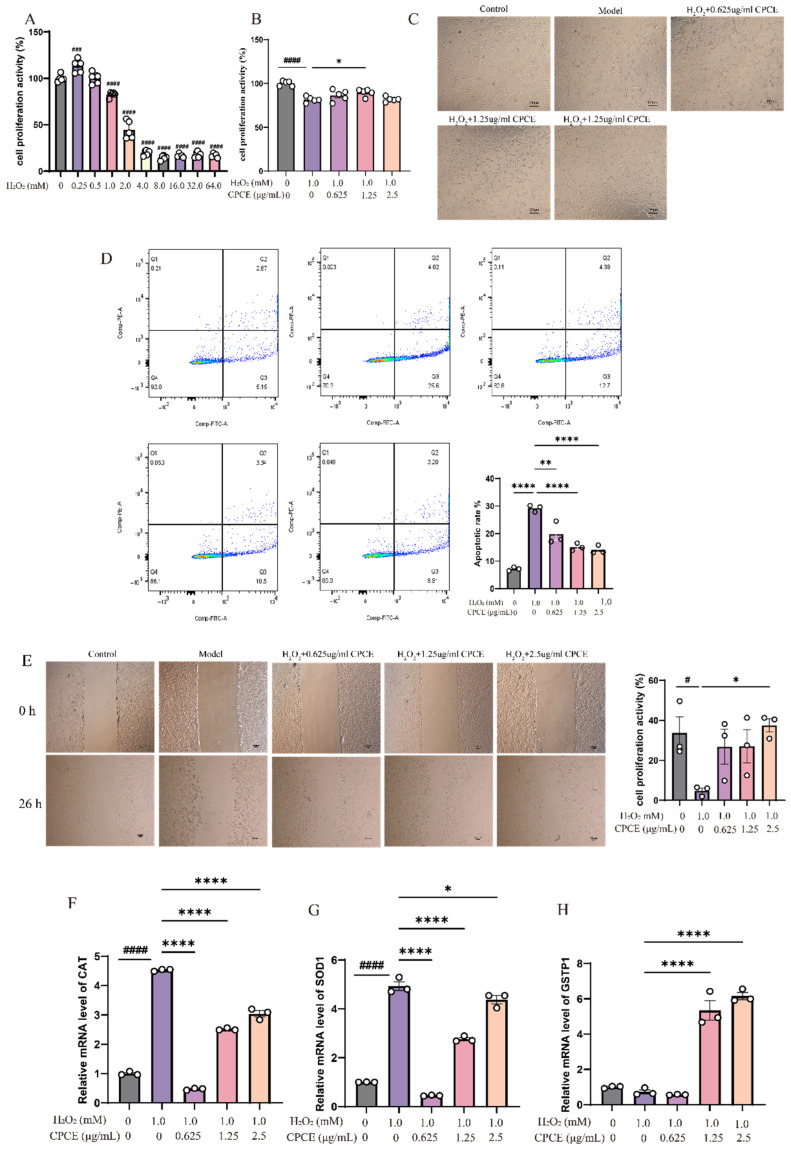
Effects of CPCE on H_2_O_2_-induced injury and oxidative stress-related endpoints in CRFK cells. (**A**) Effect of H_2_O_2_ on the survival rate of CRFK cells (*n* = 5). (**B**) Effect of CPCE on the survival rate of H_2_O_2_-treated CRFK cells (*n* = 5). (**C**) Morphological observation of CRFK cells (×10 magnification). (**D**) Effect of CPCE on the apoptosis rate of H_2_O_2_-treated CRFK cells (*n* = 3) (**E**) Effect of CPCE on the wound closure of H_2_O_2_-treated CRFK cells (×10 magnification). (images acquired at 0 and 26 h, *n* = 3). (**F**–**H**) Effect of CPCE on the mRNA expression levels of oxidative stress-related genes *CAT, SOD1, GSTP1* in H_2_O_2_-induced CRFK cells, (**F**) *CAT*; (**G**) *SOD1*; (**H**) *GSTP1*, (*n* = 3). ^#^ *p* < 0.05, ^###^ *p* < 0.001, ^####^ *p* < 0.0001 vs. control, * *p* < 0.05, ** *p* < 0.01, **** *p* < 0.0001 vs. model.

As illustrated in Figure 5A, treatment with 0.25 and 0.5 mM H_2_O_2_ did not result in a statistically significant inhibition of cell proliferation. In contrast, treatment with 1.0 mM H_2_O_2_ for 2 h led to a significant reduction in cell proliferation activity (*p* < 0.0001), indicating a pronounced reduction in cellular proliferative activity under H_2_O_2_ exposure.

#### 3.3.2. Effect of CPCE on the Proliferation Activity of H_2_O_2_-Treated CRFK Cells

As illustrated in Figure 5B, after 2 h exposure to H_2_O_2_ followed by 24 h incubation with CPCE, the administration of varying concentrations of CPCE to H_2_O_2_-treated CRFK cells resulted in enhanced cell viability compared to the model group. Specifically, treatments at lower concentrations of 0.625 and 2.5 μg/mL exhibited minimal impact on cell proliferation, with no statistically significant differences observed. In contrast, the 1.25 μg/mL treatment group demonstrated a markedly significant effect (*p* < 0.05). These findings suggest that CPCE confers a protective effect on the proliferative capacity of H_2_O_2_-treated CRFK cells.

#### 3.3.3. Effect of CPCE on the Morphology of H_2_O_2_-Treated CRFK Cells

As illustrated in Figure 5C, after 2 h H_2_O_2_ exposure treatment, cell morphology exhibited a clear group-dependent change. In the control group, CRFK cells grew adherently, were in good condition, evenly distributed, and showed no obvious damage, with spindle-shaped or oval morphology. In contrast, the H_2_O_2_-treated group displayed significant morphological changes, characterized by cell deformation, weakened adhesion, and enlarged intercellular spaces, suggesting that hydrogen peroxide induced cellular injury. In the groups treated with different concentrations of CPCE, the cells exhibited good morphology, growth state, and extension, indicating that the extracts were associated with improved cell morphology. Overall, CPCE significantly alleviated hydrogen peroxide-induced cell damage within a certain dose range, demonstrating a protective effect in this in vitro model.

#### 3.3.4. Effect of CPCE on Apoptosis in H_2_O_2_-Treated CRFK Cells

As shown in Figure 5D, treatment with 1.0 mM H_2_O_2_ significantly increased the apoptosis rate of CRFK cells relative to the control group (*p* < 0.0001), indicating that oxidative stress induced marked apoptotic injury. After CPCE intervention, the total apoptosis rate was reduced at all tested concentrations. Among them, the 0.625 μg/mL group showed a significant decrease compared with the model group (*p* < 0.01), whereas the 1.25 and 2.5 μg/mL groups showed more significant reductions (both *p* < 0.0001). Representative flow-cytometry plots further demonstrated that CPCE reduced the proportion of apoptotic cells induced by H_2_O_2_. These findings suggest that CPCE exerts a protective effect against H_2_O_2_-induced apoptosis in CRFK cells.

#### 3.3.5. Effect of CPCE on the Scratch Wound Healing Rate of H_2_O_2_-Treated CRFK Cells

The scratch healing rate of cells in the model group was significantly lower compared to the control group, while the healing rate of H_2_O_2_-treated CRFK cells was increased in the 0.625 and 1.25 μg/mL CPCE-treated groups. The 2.5 μg/mL treatment group notably accelerated the scratch healing rate in H_2_O_2_-treated CRFK cells (*p* < 0.05), indicating that CPCE can enhance scratch wound closure in these cells, as demonstrated in Figure 5E. As cell proliferation was not experimentally inhibited, wound closure may reflect contributions from both cell migration and proliferation.

#### 3.3.6. Effect of CPCE on the mRNA Expression Levels of Oxidative Stress-Related Genes in H_2_O_2_-Treated CRFK Cells

As illustrated in Figure 5F–H, following a 2 h exposure to H_2_O_2_ and subsequent incubation with varying concentrations of CPCE for 24 h, the qPCR analysis demonstrated differential modulation of gene expression. Relative to the control group, the group treated with H_2_O_2_ exhibited a significant upregulation in mRNA levels of oxidative stress-responsive genes, specifically CAT and SOD1 (*p* < 0.0001), whereas GSTP1 mRNA expression displayed a downward trend. Notably, as the concentrations of CPCE decreased, there was a significant reduction in the mRNA levels of CAT and SOD1 (*p* < 0.05), with the most pronounced effect observed in the 0.625 μg/mL group. Moreover, the expression of GSTP1 mRNA exhibited a biphasic response to CPCE treatment. A significant induction was observed at the intermediate and highest concentrations (1.25 and 2.5 μg/mL).

### 3.4. Effect of CPCE on Fibrosis-Related Responses in PA-Induced CRFK Cells

#### 3.4.1. Effect of Different Concentrations of PA on the Viability of CRFK Cells

**Figure 6:**
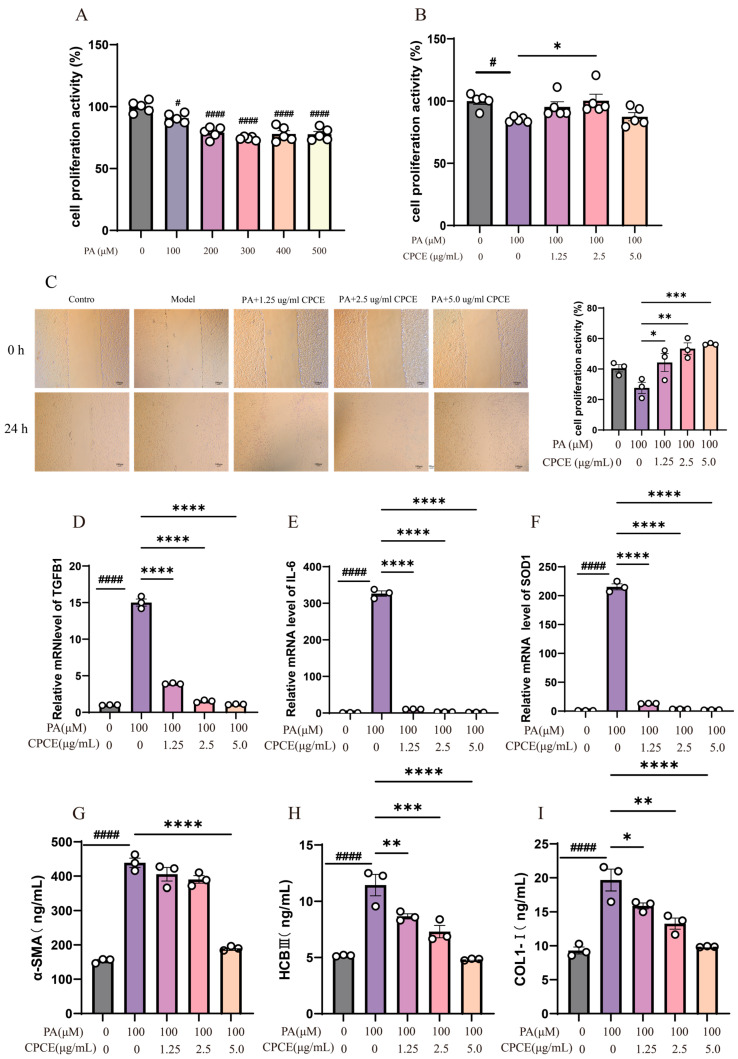
Effects of CPCE on PA-induced injury and fibrosis-related endpoints in CRFK cells. (**A**) Effect of PA on the survival rate of CRFK cells, *n* = 5. (**B**) Effect of CPCE on the survival rate of PA-induced CRFK cells, *n* = 5, (**C**) Effect of CPCE on scratch wound closure in PA-induced CRFK cells. (×10 magnification), *n* = 3, (**D**–**F**) Effect of CPCE on the mRNA expression levels of *TGFB1*, *IL-6*, and *SOD1* in PA-induced CRFK cells, *n* = 3, (**G**-**I**) Effect of CPCE on the levels of *α-SMA*, *COL I*, and HCBIII in PA-induced CRFK cells, (G) α-SMA; (H) HCB III; (I) COL I, *n* = 3. ^#^ *p* < 0.05, ^####^ *p* < 0.0001 vs. control, * *p* < 0.05, ** *p* < 0.01, *** *p* < 0.001, **** *p* < 0.0001 vs. model.

As depicted in Figure 6A, PA treatment resulted in a dose-dependent decline in cell proliferation activity. Relative to the control group, exposure to 100 μM PA significantly diminished cell viability (*p* < 0.05). In contrast, concentrations of 200, 300, 400, and 500 μM PA exerted a substantially more pronounced inhibitory effect (*p* < 0.0001). The progressive decline in cell proliferation activity with increasing PA concentrations underscores a distinct dose-dependent cytotoxic effect of PA on the cells.

#### 3.4.2. Effect of CPCE on the Proliferation Activity of PA-Induced CRFK Cells

As illustrated in Figure 6B, exposure to 100 μM PA resulted in a significant decrease in cell proliferation activity (*p* < 0.05). However, the introduction of CPCE led to a partial restoration of cell viability. Among the concentrations evaluated, the 2.5 μg/mL combination notably enhanced cell viability compared to the PA group (*p* < 0.05), whereas the 1.25 and 5.0 μg/mL concentrations did not yield a statistically significant improvement. Collectively, these results suggest that CPCE exerts a protective effect against PA-induced reductions in cell viability, with the most substantial effect observed at the 2.5 μg/mL concentration.

#### 3.4.3. Effect of CPCE on the Scratch Wound Healing Rate of PA-Induced CRFK Cells

As illustrated in Figure 6C, the 24 h wound-healing assay demonstrated a significant reduction in the cell migration rate in the group treated with 100 μM PA compared to the control group (*p* < 0.05). This finding suggests that PA has a pronounced inhibitory effect on the migratory capacity of CRFK cells. Furthermore, co-treatment with 100 μM PA and varying concentrations of CPCE resulted in differential degrees of recovery in cell migration relative to the PA-treated group. The 1.25 μg/mL combination notably enhanced the migration rate (*p* < 0.05), while the 2.5 and 5.0 μg/mL combinations had a more pronounced pro-migratory impact, with migration nearing or slightly surpassing control levels. In general, CPCE alleviated PA-induced impairment of scratch wound closure in a concentration-dependent manner. As cell proliferation was not experimentally inhibited, the observed wound closure may reflect both migration and proliferation.

#### 3.4.4. Effect of CPCE on the Gene Expression of TGFB1, SOD1, and IL-6 in PA-Induced CRFK Cells

As illustrated in Figure 6D–F, PA significantly increased the mRNA expression levels of the fibrosis-associated marker TGFB1, the pro-inflammatory cytokine gene *IL-6*, and the antioxidant enzyme *SOD1* compared to the control group (all *p* < 0.0001). This suggests that PA effectively increased *TGFB1*, *IL-6*, and *SOD*1 mRNA expression under these conditions. Conversely, when CPCE was introduced alongside PA, there was a notable downregulation in the expression of all three genes. At concentrations of 1.25, 2.5, and 5.0 μg/mL, the mRNA expression levels of *TGFB1*, *IL-6*, and *SOD1* were significantly decreased from the elevated levels induced by PA, approaching those observed in the control group. These differences were statistically significant, with all *p*-values being less than 0.001.

#### 3.4.5. Effect of CPCE on the Levels of α-SMA, COL I and HCBIII in PA-Induced CRFK Cells

As illustrated in Figure 6G–I, PA treatment significantly increased the levels of α-SMA, COL I, and HCBIII compared with the control group (all *p* < 0.0001). Conversely, co-treatment with extracts of CPCE significantly reduced these fibrosis-related markers relative to the PA model group, with more pronounced effects observed at 2.5 and 5.0 μg/mL. These results suggest that CPCE attenuated PA-induced fibrosis-related marker responses in CRFK cells under the present in vitro conditions.

## 4. Discussion

In this study, CPCE showed multi-faceted protection to CRFK cells by attenuating inflammation and apoptosis, modulating antioxidant responses and scratch wound closure, and modulating fibrosis-related responses. In addition to improving cell viability and wound closure under oxidative stress conditions, CPCE also reduced H_2_O_2_-induced apoptosis in CRFK cells, further supporting its cytoprotective effect against oxidative injury. These processes are intricately linked in the pathogenesis of chronic kidney disease, where sustained inflammatory activation and oxidative stress culminate in tubular injury and interstitial fibrosis [43,44,45]. Consequently, our findings suggest a multi-modal renoprotective effect of CPCE, which is consistent with effects on multiple injury-associated endpoints rather than a singular pathway. In addition, the preliminary HPLC fingerprint analysis of three independent batches of CPCE showed generally similar chromatographic profiles, providing supportive evidence of acceptable batch-to-batch consistency at the fingerprint level for the material used in the present study.

LPS stimulates the TLR4/NF-κB signaling pathway, leading to the release of several inflammatory mediators, which causes damage and apoptosis in renal cells [46,47]. Flow cytometry analysis demonstrated that the extracts significantly inhibited LPS-induced apoptosis in CRFK cells, suggesting that the extract may contribute to mitigating renal cell injury under these in vitro conditions. Natural bioactive compounds, especially those that modulate the TLR4/NF-κB signaling pathway, have been demonstrated to play a pivotal role in the regulation of cellular inflammation and apoptosis [27,48,49]. CPCE significantly downregulated the expression of inflammatory cytokines, including *TNF-α* and *IL-6*, thereby attenuating the inflammatory response induced by LPS. These findings align with previous research demonstrating that PAE attenuates LPS-induced inflammation through the suppression of inflammatory cytokine expression [26,50]. The *Bax/Bcl-2* ratio is a critical determinant of apoptosis. The extracts generally induced a shift in apoptosis-related gene expression towards an anti-apoptotic profile, as evidenced by consistent changes in *Bcl-2* and other markers compared to the model group. Interestingly, Bax demonstrated a non-monotonic response, with an increase observed at 5.0 μg/mL despite decreases at lower concentrations. This phenomenon may indicate concentration-dependent activation of distinct regulatory pathways and/or differential target engagement characteristic of multi-constituent extracts. Consequently, the increase in Bax at the highest concentration warrants cautious interpretation, and the effective concentration range should be further delineated through protein-level *Bax/Bcl-2* measurements and functional apoptosis assays [51]. RT-qPCR results showed that the extracts significantly altered the transcript levels of key apoptosis-related genes, including Bax and *Bcl-2*. Collectively, these findings indicate that CPCE treatment may mitigate LPS-induced cellular injury in part through coordinated changes in inflammation-associated and apoptosis-associated responses [33,52]. Notably, because the present study primarily assessed transcript-level changes, the absence of protein-level analyses (e.g., Western blotting or immunofluorescence) and functional apoptosis assays (e.g., caspase activity) limits definitive conclusions regarding apoptosis pathway modulation. The changes in inflammation, apoptosis, and fibrosis markers suggest that TLR4/NF-κB and TGF-β1 signaling may be involved in CPCE’s protective effects. However, since pathway activation was not directly assessed, these conclusions are inferential and need further validation through specific assays.

Furthermore, this study conducted a comprehensive evaluation of the antioxidant properties of CPCE through a series of experiments. The findings revealed that the extracts exerted significant protective effects on cell viability, morphology, scratch wound closure, and oxidative stress–related gene expression. Specifically, in the context of cell morphology, CRFK cells exposed to the H_2_O_2_ treatment group displayed notable morphological alterations, such as cell deformation, diminished adhesion, and enlarged intercellular spaces, indicative of considerable structural damage induced by oxidative stress [53]. In the treatment group, the cells demonstrated an even distribution and exhibited a morphology that closely resembled normal characteristics, thereby providing further validation of the protective effect of the extract on cellular integrity. These results indicated that the extract effectively alleviated morphological damage caused by oxidative stress and significantly improved the overall health status of the cells. In the scratch assay, which assessed the effect of the extract on cell migration, we found that CPCE significantly increased scratch wound closure of H_2_O_2_-treated CRFK cells, especially at a lower concentration of 2.5 μg/mL, where wound closure rates were markedly enhanced (*p* < 0.05). Because cell proliferation was not experimentally inhibited, wound closure may reflect contributions from both cell migration and proliferation. Furthermore, analogous studies have demonstrated that natural extracts derived from insects are instrumental in promoting cell migration and tissue repair [21,54]. *SOD1* and *CAT*, as core antioxidant enzymes, scavenge excess ROS, thereby alleviating oxidative stress-induced cellular damage. GSTP1 plays a key role in antioxidant stress by participating in detoxification processes, further boosting the cell’s resistance to oxidative damage [55,56,57]. CPCE consistently modulated the expression of *CAT*, *SOD1*, and *GSTP1*, indicating its potential to normalize the dysregulation of oxidative stress-responsive genes induced by H_2_O_2_, thereby mitigating the oxidative stress burden in CRFK cells.

In terms of anti-fibrosis, this study evaluated the effects of CPCE through multiple experiments. Fibrosis is a key pathological process in various kidney diseases, especially CKD, characterized by excessive collagen deposition and expansion of the renal tubular interstitium. Therefore, anti-fibrotic treatment has become an important strategy for improving kidney function and delaying disease progression [58,59]. Firstly, in the scratch assay, the extract significantly promoted the scratch wound closure of PA-induced CRFK cells, indicating its potential association with cellular recovery processes. As noted above, because proliferation was not inhibited, this assay reflects wound closure rather than complete tissue repair or regeneration. Cell migration is involved in tissue repair-related processes; however, in the present study, the scratch assay was used only as an indicator of wound closure behavior and not as a direct readout of fibrosis reversal [60]. In the present PA-induced model, PA exposure was associated with increased TGFB1 expression, together with changes in *IL-6* and *SOD1*, suggesting activation of fibrosis-related, inflammatory, and oxidative stress–related responses under the present experimental conditions. TGF-β1 is a key signaling molecule in the fibrosis process, capable of inducing fibroblast activation and collagen deposition, making it an important target in various kidney diseases [61]. IL-6, as a pro-inflammatory cytokine, also plays a crucial role in the progression of fibrosis by promoting immune responses and inflammatory reactions, thereby driving the formation of fibrosis [62]. SOD1, as a key antioxidant enzyme, can scavenge excess ROS, thereby protecting cells from damage induced by oxidative stress and playing a crucial protective role in the occurrence and progression of fibrosis [55]. Our experimental results suggested that CPCE may alleviate renal fibrosis by inhibiting the expression of fibrosis-related factors such as TGF-β1, as well as modulating inflammation- and oxidative stress–related genes such as *IL-6* and *SOD1*, thereby potentially slowing fibrosis-associated responses in renal cells. In the present study, classical structural fibrosis markers, including α-SMA, collagen I, and collagen III, were additionally evaluated to strengthen the assessment of fibrosis-related responses. The coordinated modulation of these markers, together with *TGFB1*, supports an antifibrotic profile at the cellular level. Nevertheless, as these findings are derived from in vitro experiments, further validation in vivo models remains necessary to confirm functional antifibrotic efficacy.

Our findings align with the hypothesis that CPCE exhibits a multi-pathway renoprotective profile. CPCE appears to attenuate LPS-induced inflammatory and apoptosis-associated responses, enhance antioxidant response-related outcomes, and facilitate scratch wound closure under H_2_O_2_-induced oxidative stress. Additionally, CPCE modulates fibrosis-related markers, including TGFB1, α-SMA, collagen I, and collagen III/HCBIII, within the PA-induced model. Consequently, they concurrently address inflammation, oxidative stress, and fibrosis-related endpoints, suggesting potential utility compared to single-target agents in vitro as functional ingredients for the prevention or adjunctive treatment of feline kidney disease. Given the in vitro nature of the present work, any translational application should be considered prospective and will require in vivo validation, toxicological safety assessment, evaluation of bioavailability, and extract standardization.

However, several limitations must be acknowledged: all experiments were conducted in vitro using a single CRFK cells, and a crude CPCE was utilized without isolating specific active constituents or directly comparing the combination with each single extract. Although we added a preliminary HPLC fingerprint analysis and observed generally consistent chromatographic profiles across three CPCE batches, this assessment provides only fingerprint-level batch consistency and does not constitute full chemical characterization or marker-based standardization. Furthermore, no animal or clinical studies, or pathway-specific inhibition/gene-silencing approaches, were undertaken to verify in vivo efficacy, safety, or the causal involvement of TLR4/NF-κB and TGF-β1 signaling. Future studies should therefore focus on optimizing dosing and the mixing ratio of the two extracts, further characterizing and standardizing their chemical profiles and quality markers, and validating these renoprotective effects and mechanisms in well-designed in vivo models to facilitate translation into practical applications in feline nutrition and therapeutics. As CPCE is a crude combined extract, its biological activity may result from the combined effects of multiple constituents rather than a single compound. Although inosine and cinnamic acid were identified as major constituents in the present study, further phytochemical characterization and bioactivity-guided fractionation will still be needed to clarify their relative contributions and to further explore potential synergistic interactions among the different components of CPCE.

## 5. Conclusions

In summary, CPCE demonstrated modulatory effects on inflammation, oxidative stress, and fibrosis-related responses in CRFK cells under in vitro conditions, indicating potential cytoprotective properties in a feline renal cell model. Insect-derived extracts are abundant in diverse bioactive compounds that may possess functional properties in vitro. This study offers preliminary experimental evidence supporting the further exploration of insect-derived extracts as potential functional ingredients. However, their clinical, nutritional, or pharmaceutical applicability necessitates additional validation, including in vivo efficacy studies, safety and toxicological evaluations, bioavailability assessments, and extract characterization and standardization.

## Figures and Tables

**Table 1:** Anti-Inflammation-Related Primer information.

Gene	Primer	Sequence
*GAPDH*	Forward	CAAATGGGGTGATGCTGGTGCT
	Reverse	CGGTCTTCTGGGTGGCGGTGAT
*TNF-α*	Forward	CCTGCTGCACTTTGGAGTGA
	Reverse	GCTTCGGGGTTTGCTACTACA
*IL-6*	Forward	TGGGTTCAATCAGGAGACCT
	Reverse	TTCTACGGTTGGGACAGGGA
*Bax*	Forward	TTCAGGGTTTCATCCAAGATC
	Reverse	AAAGTAGAAGAGGGCAACGAC
*Bcl-2*	Forward	CTTCAGGGATGGCGTGAACT
	Reverse	GTTCCACAAAGGCATCCCAG

**Table 2:** Oxidative Stress-Related Primer information.

Gene	Primer	Sequence
*CAT*	Forward	ACGCCTGTGTGAGAACATTG
	Reverse	TCACTGAAGTTCTTGACCG
*GSTP1*	Forward	TCGCAGCAAATACATCACCC
	Reverse	GTCTCGAAAGGCTTCAGGTG
*SOD1*	Forward	CATCATTGGCCGCACGAT
	Reverse	ATGACACCACAAGCCAAACG

**Table 3:** Fibrosis-Related Primer information.

Gene	Primer	Sequence
*TGFB1*	Forward	GTGGACATCAACGCAGGGTTCAG
	Reverse	CCGCACGCAGCAGTTCTTCTC

## Data Availability

The original contributions presented in this study are included in the article/Supplementary Materials. Further inquiries can be directed to the corresponding authors.

## Supplementary Materials

The following supporting information can be downloaded at: https://www.mdpi.com/article/10.3390/vetsci13040317/s1, Table S1: Compensation matrix for flow-cytometric apoptosis analysis in LPS-induced CRFK cells; Figure S1: Gating strategy for flow-cytometric apoptosis analysis in LPS-induced CRFK cells; Table S2: Compensation matrix for flow-cytometric apoptosis analysis in H2O2-induced CRFK cells; Figure S2: Gating strategy for flow-cytometric apoptosis analysis in H2O2-induced CRFK cells.

## Abbreviations

CCME, *Cybister chinensis* extracts; PAE, *Periplaneta americana* residue extracts; CRFK, Crandell-Rees Feline Kidney; LPS, lipopolysaccharide; H_2_O_2_, hydrogen peroxide; PA, palmitic acid; CPCE, combined extract of PAE and CCME; PBS, phosphate-buffered saline; PI, propidium iodide; FSC, forward scatter; SSC, side scatter; ER, endoplasmic reticulum; FFA, free fatty acid; ROS, reactive oxygen species; NF-κB, nuclear factor kappa B; STAT3, signal transducer and activator of transcription 3; TFA, trifluoroacetic acid; COL I, collagen type I; HCBIII, feline collagen type III.
