# Supplementary material for: Protective Effects Assessment of Combined Extracts from Periplaneta americana Residues and Cybister chinensis Motschulsky on Feline Renal Cells: In Vitro Evidence Related to Inflammation, Oxidative Stress, and Fibrosis
Source: Vet Sci. 2026 Mar 26;13(4):317. doi: 10.3390/vetsci13040317 (PMC13119821; doi:10.3390/vetsci13040317)
Supplement: Supplementary file 1 [file vetsci-13-00317-s001.zip › vetsci-4095529-supplementary.pdf]

Supplementary Materials

Table S1. Compensation matrix for flow-cytometric apoptosis analysis in LPS-induced CRFK cells.

Detector	FITC-A	PE-A
FITC-A	100.0	51.9
PE-A	0.5	100

Compensation matrix used for Annexin V-FITC/PI apoptosis analysis in the LPS-induced CRFK cell model.

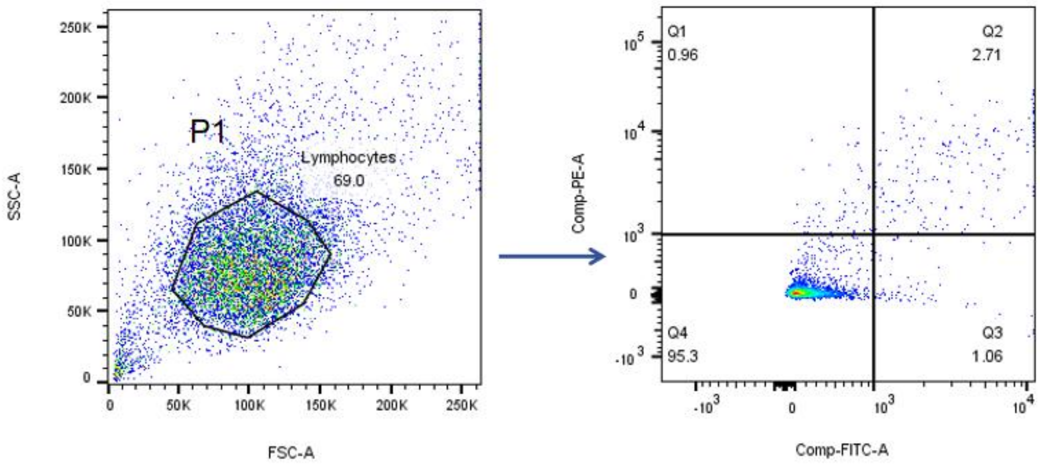

The P1 gate ensures that the cells are in the gated area.

Cell populations were classified based on the Annexin V/PI double-parameter plot.

Figure S1. Gating strategy for flow-cytometric apoptosis analysis in LPS-induced CRFK cells.

Gating strategy used for Annexin V-FITC/PI flow-cytometric apoptosis analysis in the LPS-induced CRFK cell model. Debris and non-cellular events were excluded using forward scatter (FSC) and side scatter (SSC) parameters. Apoptotic cell populations were then quantified by quadrant analysis based on Annexin V-FITC and PI staining. Annexin V^{-}/PI^{-} cells were defined as viable cells, Annexin V^{+}/PI^{-} cells as early apoptotic cells, Annexin V^{+}/PI^{+} cells as late apoptotic cells, and Annexin V^{-}/PI^{+}

cells as necrotic cells. The total apoptosis rate was calculated as the sum of early and late apoptotic cells.

Table S2. Compensation matrix for flow-cytometric apoptosis analysis in H_2O_2-induced CRFK cells.

Detector	FITC-A	PE-A
FITC-A	100.0	18.5
PE-A	3.5	100

Compensation matrix used for Annexin V-FITC/PI apoptosis analysis in the H_2O_2-induced CRFK cell model.

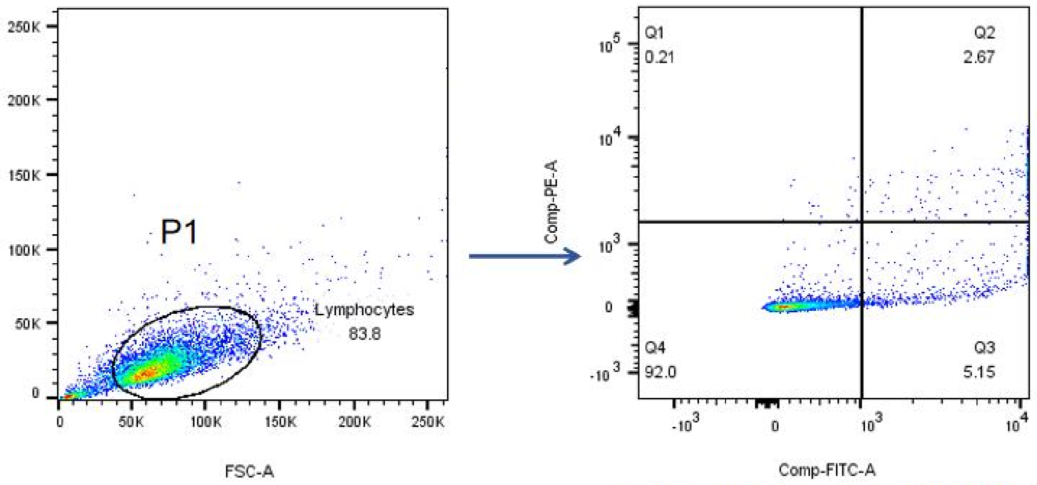

The P1 gate ensures that the cells are in the gated area.

Cell populations were classified based on the Annexin V/PI double-parameter plot.

Figure S2. Gating strategy for flow-cytometric apoptosis analysis in H_2O_2-induced CRFK cells.

Gating strategy used for Annexin V-FITC/PI flow-cytometric apoptosis analysis in the H_2O_2-induced CRFK cell model. Debris and non-cellular events were excluded using forward scatter (FSC) and side scatter (SSC) parameters. Apoptotic cell populations were then quantified by quadrant analysis based on Annexin V-FITC and PI staining. Annexin V^-/PI^- cells were defined as viable cells, Annexin V^+/PI^- cells as early apoptotic cells, Annexin V^+/PI^+ cells as late apoptotic cells, and Annexin V^-/PI^+

cells as necrotic cells. The total apoptosis rate was calculated as the sum of early and late apoptotic cells.
